# Gossypol from Cottonseeds Ameliorates Glucose Uptake by Mimicking Insulin Signaling and Improves Glucose Homeostasis in Mice with Streptozotocin-Induced Diabetes

**DOI:** 10.1155/2018/5796102

**Published:** 2018-10-28

**Authors:** Md Badrul Alam, Hongyan An, Jeong-Sic Ra, Ji-young Lim, Seung-Hyun Lee, Chi-Yeol Yoo, Sang-Han Lee

**Affiliations:** ^1^Department of Food Science and Biotechnology, Graduate School, Kyungpook National University, Daegu 41566, Republic of Korea; ^2^Food and Bio-Industry Research Institute, Inner Beauty/Antiaging Center, Kyungpook National University, Daegu 41566, Republic of Korea

## Abstract

Glucose absorption from the gut and glucose uptake into muscles are vital for the regulation of glucose homeostasis. In the current study, we determined if gossypol (GSP) reduces postprandial hyperglycemia or enhances glucose uptake; we also investigated the molecular mechanisms underlying those processes *in vitro* and *in vivo*. GSP strongly and concentration dependently inhibited *α*-glucosidase by functioning as a competitive inhibitor with IC_50_ value of 0.67 ± 0.44. GSP activated the insulin receptor substrate 1 (IRS-1)/protein kinase B (Akt) signaling pathways and enhanced glucose uptake through the translocation of glucose transporter 4 (GLUT4) into plasma membrane in C2C12 myotubes. Pretreatment with a specific inhibitor attenuated the *in vitro* effects of GSP. We used a streptozotocin-induced diabetic mouse model to assess the antidiabetic potential of GSP. Consistent with the *in vitro* study, a higher dose of GSP (2.5 mg/kg^−1^) dramatically decreased the postprandial blood glucose levels associated with the upregulated expressions of GLUT4 and the IRS-1/Akt-mediated signaling cascade in skeletal muscle. GSP treatment also significantly boosted antioxidant enzyme expression and mitigated gluconeogenesis in the liver. Collectively, these data imply that GSP has the potential in managing and preventing diabetes by ameliorating glucose uptake and improving glucose homeostasis.

## 1. Introduction

**Figure 1:**
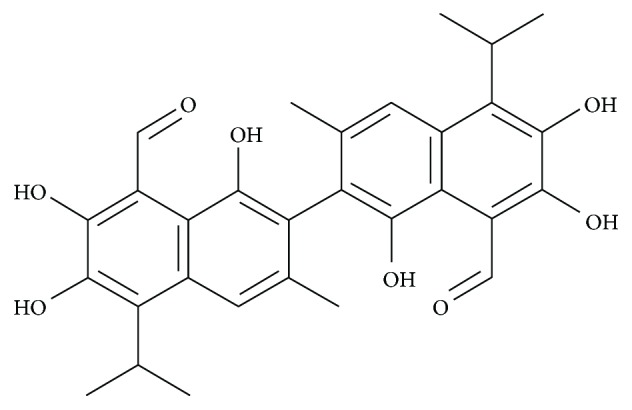
Chemical structure of gossypol (GSP) in cottonseed extracts.

Gossypol (GSP) is a lipid-soluble polyphenolic bis-sesquiterpene. It is extracted from cottonseeds as a racemic mixture owing to hampered rotation around the binaphthyl bond (Figure 1, Supplementary Figure S1). GSP acts as nonsteroidal contraceptive by inhibiting energy metabolism-related enzymes in sperm and spermatogenic cells, which suppresses the production and motility of sperm [1, 2]. It also has antiviral [3], antiparasitic [4], and inflammation-inhibitory properties [5] and has been shown to obstruct rat liver microsomal peroxidation and prevent damage to supercoiled plasmid DNA induced by the Fenton reaction [6]. Furthermore, (−)-gossypol is more cytotoxic than (+)-gossypol and/or racemic gossypol at lower concentrations in various cancer cells [7]. GSP suppresses the key nuclear enzymes involved in DNA replication and repair and induces apoptosis by activating caspase pathways or suppressing NF-*κ*B activity [8–10].

Diabetes mellitus (DM) is a complex, polygenic metabolic disease. It appears either when the pancreas is incapable of producing the optimal level of insulin or when the body develops insulin resistance, resulting in elevated blood glucose levels [11]. It is predicted that, globally, the number of DM patients are evoked to 439 million by 2030 and among them type 2 diabetes mellitus (T2-DM) will reach 90%. It is well known that the production of low insulin or the development of insulin resistance owing to alteration of genetic and/or epigenetic factors is intimately associated with the development of T2-DM [12]. Insulin resistance is a pivotal feature of T2-DM and is initiated by reduced glucose uptake into skeletal muscles and adipose tissues. Approximately 75% of glucose is absorbed by insulin-stimulated skeletal muscles, and glucose transport is viewed as the rate-limiting step in primary glucose disposal and utilization [13, 14]. It is thought that insulin aids to translocate glucose by glucose transporter 4 (GLUT4) into the cell membrane of skeletal muscle and enhance the postprandial glucose uptake. Reduced intracellular GLUT4 traffic into the cell membrane is one of the major causes of T2-DM [13].

However, the enhancement of glucose uptake in skeletal muscle is regulated by two major signaling pathways. One is recognized as an insulin-dependent pathway, which implies the activation of insulin receptor (IR) by insulin, and initiates signaling pathways such as IR substrate (IRS), phosphoinositide 3-kinase (PI3K), Akt, atypical protein kinase C (aPKC), and GLUT4 translocation, resulting in enhanced glucose uptake by skeletal muscle [15]. Consequently, glucose homeostasis is closely associated with optimal insulin production in *β*-cells and its sensitivity in skeletal muscle or adipose tissue. On the other hand, insulin-independent pathway, which is arbitrated by 5′-adenosine monophosphate-activated protein kinase (AMPK), plays a focal function in metabolic regulation and the homeostasis of cellular energy balance [16].

The escalating number of diabetics in the world necessitates the development of new and effective therapy options. Insulin therapy is currently among the best solutions available for the treatment of both types of diabetes (types 1 and 2). Accumulating research suggested that natural products and some small synthetic molecules can activate the insulin signaling pathway and boost glucose uptake in cultured cells and in animal diabetic models [17]. There is a rising trend towards natural plant remedies in modern clinical medicine for treating T2-DM due to having acceptable efficacy and limited reverse effects. Such remedies are receiving increasing interest because they are safe [18].

During screening for antidiabetic agents from natural products, in the current study, we scrutinized the antidiabetic capability of GSP on glucose uptake in C2C12 myotubes and attempted to elucidate the core molecular mechanism. We also determined whether GSP has the potential to improve glucose homeostasis in mice with streptozotocin- (STZ-) induced diabetes.

## 2. Materials and Methods

### 2.1. *α*-Glucosidase Inhibitory Assay

The *α*-glucosidase inhibitory activity of GSP was measured according to the method defined by Zhao et al. [16]. Briefly, 2 *μ*L aliquots of predetermined concentration of GSP was mixed with 0.2 U/mL *α*-glucosidase in 0.1 M sodium phosphate buffer (pH 7.0). Subsequently, pNPG (2 mM), acting as a substrate, was added to the solution to start enzyme-substrate reactions, which were allowed to proceed for 30 min at 37°C. The *α*-glucosidase inhibition activity was calculated spectrophotometrically at 405 nm using a 96-well plate on a microplate reader (PerkinElmer Wallac Victor3, MA, USA). Acarbose served as a positive control, and following equation was adopted to evaluate the percent inhibition:

(1) $$\begin{array}{c} \alpha -Glucosidase\;inhibitory\;activity\left(\%\text{ of }inhibition\right)=\frac{{Abs}_{control}-{Abs}_{sample}}{{Abs}_{control}}\times 100. \end{array}$$

The inhibition kinetics of GSP on *α*-glucosidase was determined by preparing a series of sample solutions with various concentrations of substrate and GSP. The mode of inhibition of GSP was deduced from a Lineweaver–Burk plot and computed the Km (dissociation constant) and *V*_max_ (maximum reaction velocity) of the enzyme [19].

### 2.2. Cell Culture, Cell Differentiation, and Glucose Uptake Assay

C2C12 cells (American Type Culture Collection, Manassas, VA, USA) were cultured at 37°C in Dulbecco's modified Eagle's medium (DMEM) supplemented with 10% fetal bovine serum (FBS) and streptomycin-penicillin (100 *μ*g/mL and 100 U/mL, respectively; HyClone, Mordialloc, VIC, Australia) in a humidified 5% CO_2_ atmosphere. The cells were grown in 96-well plates (1 × 10^5^ cells/mL) with DMEM containing 10% FBS and 1% penicillin/streptomycin at 37°C in the CO_2_ incubator. When the cells reached confluence, they were subjected to differentiation in DMEM supplemented with 2% horse serum for 5 days. They were then starved in low-glucose serum-free DMEM for 24 h. Subsequently, we carried out a 2-(N-(7-nitrobenz-2-oxa-1,3-diazol-4-yl) amino)-2-deoxyglucose (2-NBDG) assay to evaluate glucose uptake [20]. Briefly, the cells were pretreated with advised concentrations of GSP and insulin (100 nM) for the indicated time interval, followed by 20 *μ*M of 2-NBDG for 1 h. After incubation, the cells were rinsed twice with cold phosphate-buffered saline (PBS), and 2-NBDG uptake was measured using a fluorometer (PerkinElmer, Wallac Victor3, MA, USA) at excitation and emission wavelengths of 490 and 535 nm, respectively.

### 2.3. Extraction of Cell Membrane Protein

The cells were rinsed with cold PBS and harvested. A cell membrane protein isolation kit (Invent Biotechnologies Inc., Eden Prairie, MN, USA) was used to extract membrane protein from the cellular components (nuclei, cytosol, and organelles) allowing to the manufacturer's instructions. A BCA Protein Assay Reagent (Pierce, Rockford, IL, USA) was applied to calculate the protein content.

### 2.4. Animal Experiments and STZ-Induced Diabetes

We used six-week-old male BALB/c mice (Samtako, Osan, Republic of Korea). The mice were housed in an organized room at 22 ± 10°C and 55 ± 5% humidity with a 12 h light/dark cycle and had *ad libitum* access to food and water. Each mouse was isolated and adapted to the laboratory environment for at least 1 week prior to the experiment, according to the guidelines specified by the Committee on Laboratory Animal Ethics, Kyungpook National University (KNU 2017-0049, Daegu, Republic of Korea). The mice were randomly divided into 5 groups, each comprising 5 animals: a normal control group (G1), an STZ-induced diabetic control group (G2), an STZ-induced diabetic plus rosiglitazone group (10 mg/kg bodyweight (b.w.)) (G3), an STZ-induced diabetic plus low-dose GSP group (1 mg/kg b.w.) (G4), and an STZ-induced diabetic plus high-dose GSP group (2.5 mg/kg b.w.) (G5). Diabetes was induced by intraperitoneally injecting the diabetic group with STZ dissolved in 50 mM citrate buffer (pH 4.5) at 75 mg/kg for 3 successive days. The G1 group was introduced with an equivalent amount of citrate buffer. After 4 days, fasting blood glucose (FBG) levels were calculated, and mice with levels ≥ 200 mg/dL were chosen for the experiment.

### 2.5. Oral Glucose Tolerance Test (OGTT)

We accomplished an OGTT after fasting the mice overnight to resolve the effects of GSP on glucose tolerance. To complete this test, we orally administered a single dose of glucose solution (1 g/kg) and GSP (1 and 2.5 mg/kg) to each mouse and measured the subsequent blood glucose levels using an ACCU-CHEK® Active glucometer (Roche Diagnostics, Mannheim, Germany) at 0, 30, 60, 90, 120, 150, and 180 min after administering the glucose solution [21].

### 2.6. Biochemical and Histological Analysis

At the end of the experimental period, the mice were sacrificed and blood samples were then gathered for biochemical estimations. We carefully harvested the major organs such as the pancreas, liver, and skeletal muscle. Parts of the liver and skeletal muscle were immediately submerged in TRIzol solution for reverse transcription-polymerase chain reaction (RT-PCR) analysis, and the residual tissue was kept in liquid nitrogen for Western blotting. The pancreases were stored in 10% formalin solution, embedded in paraffin, and stained with hematoxylin–eosin for histochemical examination.

### 2.7. Reverse Transcription-Polymerase Chain Reaction (RT-PCR)

Total RNA was isolated from the C2C12 cells using TRIzol (Ambion, Austin, TX, USA), consistent with the manufacturer's instructions. To prepare a cDNA, 2 *μ*g of total RNA was mixed with an RT-& GO Mastermix (MP Biomedicals, Seoul, Republic of Korea) and used as the PCR template. RT-PCR was performed using a PCR Thermal Cycler (Dice TP600, Takara Bio Inc., Otsu, Japan) by the various primer sequences (Supplementary Table S1). A 2% agarose gel in Tris-acetate-EDTA (TAE) buffer was used to separate the PCR products and visualized by ethidium bromide staining, and band intensity was analyzed by Image Lab™ software (version 5.2.1).

### 2.8. Preparation of Cell Lysates and Western Blotting

After treatment, the C2C12 myotubes were rinsed twice in ice-cold PBS and lysed using ice-cold radio-immunoprecipitation assay (RIPA) buffer with a phosphatase and protease inhibitor cocktail (Sigma-Aldrich, St. Louis, MO, USA). The skeletal muscle tissue was homogenized in ice-cold RIPA buffer containing protease inhibitors. The cell lysates and tissue homogenates were centrifuged at 3000 ×g for 10 min at 4°C to discard insoluble materials. Protein content was measured using BCA protein assay kit (Pierce, Rockford, IL, USA). Protein (20 *μ*g) was subjected to sodium dodecyl sulfate polyacrylamide gel electrophoresis (SDS-PAGE; 10%), transferred to nitrocellulose membranes (Whatman, Dassel, Germany), blocked with 5% nonfat milk in TBST buffer (a mixture of Tris-buffered saline and Tween 20), and blotted with each primary antibody (1 : 1000) and with the corresponding secondary antibody (1 : 5000). Protein bands were identified using a SuperSignal West Femto maximum sensitivity substrate (Thermo Fisher Scientific, Rockford, IL, USA), and bands were analyzed by Image Lab™ software (version 5.2.1).

### 2.9. Statistical Analysis

The data were subjected to one-way analysis of variance (ANOVA) and are presented as means ± SDs. The analysis was executed using GraphPad Prism 7 (GraphPad Software, La Jolla, CA, USA), and statistical significance was approved for *p* values of *p* < 0.01 or <0.05, as denoted.

## 3. Results

### 3.1. Kinetic Studies and Evaluation of *α*-Glucosidase Inhibition

**Figure 2:**
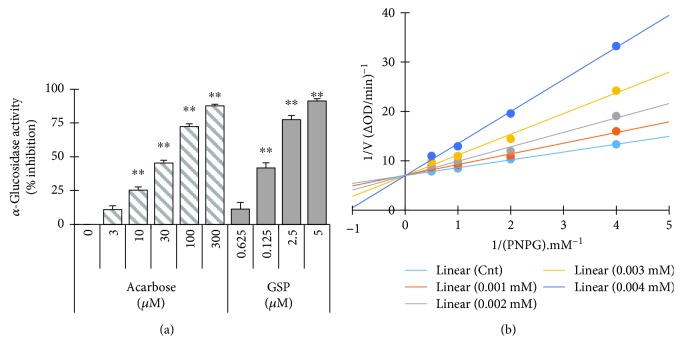
Effects of gossypol (GSP) on *α*-glucosidase activity. (a) Various concentrations of GSP or acarbose were incubated with the same units of *α*-glucosidase, and the degree of production of *p*-nitrophenol was measured spectrophotometrically at 405 nm. ^∗^ *p* < 0.05, ^∗∗^ *p* < 0.01, versus nontreated controls. (b) Lineweaver–Burk plot of *α*-glucosidase with GSP. Results are shown as mean values of 1/V, as inverses of increases in absorbance at 405 nm/min (Δ*A*_405_ per min), and as the means of three independent tests at various PNPG concentrations.

GSP inhibited *α*-glucosidase in a concentration-dependent fashion; the half maximal inhibitory concentration (IC_50_) was 0.67 ± 0.44 *μ*M. We used acarbose, a competitive *α*-glucosidase inhibitor, as a positive control (with an IC_50_ value of 46.97 ± 0.71) (Figure 2(a)). We also carried out kinetic analyses to confirm the nature of the interaction between GSP and *α*-glucosidase. Lineweaver–Burk plots were fabricated using various concentrations of GSP.  Figure 2(b) indicates that GSP exhibited typical reversible competitiveness, with a series of lines with various slopes intersecting the *y*-axis. The calculated Km and *V*_max_ are represented in Table 1.

### 3.2. GSP Stimulated Basal- and Insulin-Mediated Glucose Uptake in C2C12 Myotubes

**Figure 3:**
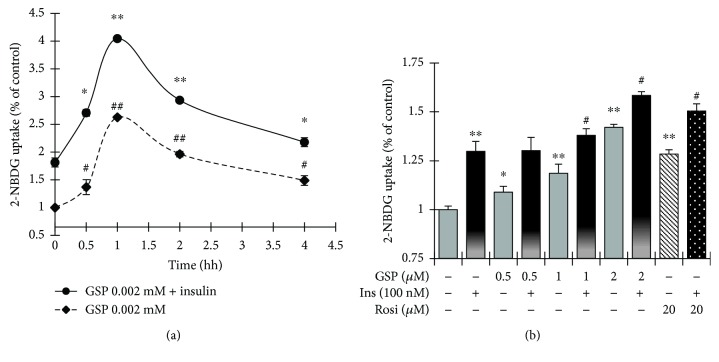
Effects of gossypol (GSP) on glucose uptake in C2C12 myotubes. (a) Time course of the effect of GSP on glucose uptake in C2C12 myotubes. Cells were treated with GSP (2 *μ*M) alone or GSP (2 *μ*M), followed by insulin (100 nM) for 30 min, and then incubated for the time periods indicated. (b) The dose-response relationship of the effects of GSP on glucose uptake on C2C12 myotubes. Cells were incubated with GSP for 2 h. Rosiglitazone (20 *μ*M) was used as a positive control. The data denote means ± SEM of three independent experiments. ^∗^ *p* < 0.05 and ^∗∗^ *p* < 0.01, versus basal glucose uptake (no insulin stimulation); ^#^ *p* < 0.05 and ^##^ *p* < 0.01, versus insulin-activated glucose uptake. Rosi: rosiglitazone.

To observe the potential of GSP on glucose uptake *in vitro*, C2C12 myotubes (insulin-sensitive mouse skeletal muscle cells) were treated with GSP at the designated time-points. As shown in Figure 3(a), 2 *μ*M GSP evoked basal- or insulin-activated glucose uptake from 0.5 h, which peaked at 1 h then gradually decreased. Therefore, we used the 1 h time-point for GSP treatment in the subsequent experiments.  Figure 3(b) shows that basal- or insulin-induced glucose uptake increased in a concentration-dependent fashion without any toxic effects (Supplementary Figure S2). At the highest concentration of GSP (2 *μ*M), the basal- and insulin-activated glucose uptake levels augmented significantly by 42.11 ± 0.21% and 58.40 ± 0.25%, respectively. Rosiglitazone—a well-known thiazolidinedione-class antidiabetic agent, used here as a positive control—also significantly amplified the basal- and insulin-induced glucose uptake levels by 28.41 ± 2.2% and 51.43 ± 4.0%, respectively.

### 3.3. GSP Promoted the Translocation of GLUT4 in C2C12 Cells

**Figure 4:**
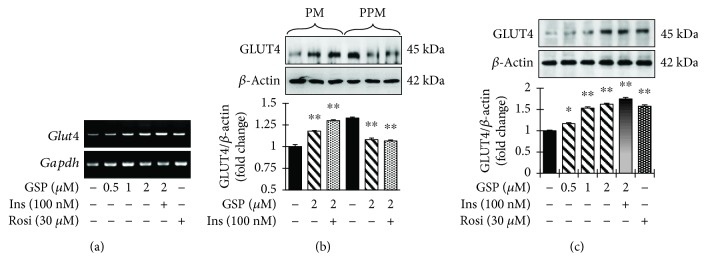
Increase of glucose uptake through the translocation of GLUT4 in C2C12 myotubes by gossypol (GSP). (a) RT-PCR analysis was carried out to measure the mRNA levels. (b, c) Subcellular membrane fractions were separated and subjected to immunoblot analysis with the indicated antibodies. Statistical data are represented in the adjacent figure. Values are shown as means ± SEM in independent triplicate. ^∗∗^ *p* < 0.01 versus no treatment. Ins: insulin, Rosi: rosiglitazone.

We used RT-PCR and Western blotting to determine whether the ability of GSP to increase glucose transport in C2C12 myotubes is associated with enhanced GLUT4 translocation, GLUT4 mRNA levels, and the amount of GLUT4 in the cell membrane and post-cell membrane fractions in the absence and presence of GSP and insulin. GSP meaningfully augmented the level of GLUT4 mRNA in a concentration-reliable fashion (Figure 4(a)). The immunoblot data (Figure 4(b)) also revealed that GSP increased GLUT4 translocation in both basal- and insulin-induced conditions. Furthermore, GSP treatment boosted GLUT4 translocation in the PM in a concentration-dependent manner (Figure 4(c)).

### 3.4. GSP Mimicked Insulin Signaling in C2C12 Cells

**Figure 5:**
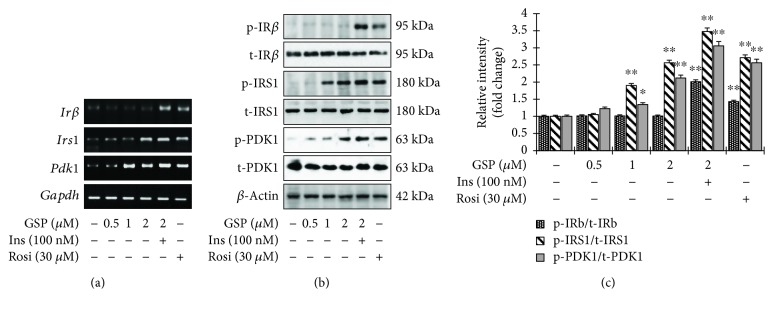
Improvement of glucose uptake via the activation of IRS-1 and its downstream signaling pathways in C2C12 myotubes by gossypol (GSP). (a) mRNA expression was analyzed by RT-PCR. (b) Total cell lysates were extracted and subjected to Western blot analysis with the designated antibodies. (c) The immunoblotting signals were quantified using densitometer. Data are shown as means ± SEM, ^∗^ *p* < 0.05 and ^∗∗^ *p* < 0.01, versus no treatment. Rosi: rosiglitazone.

**Figure 6:**
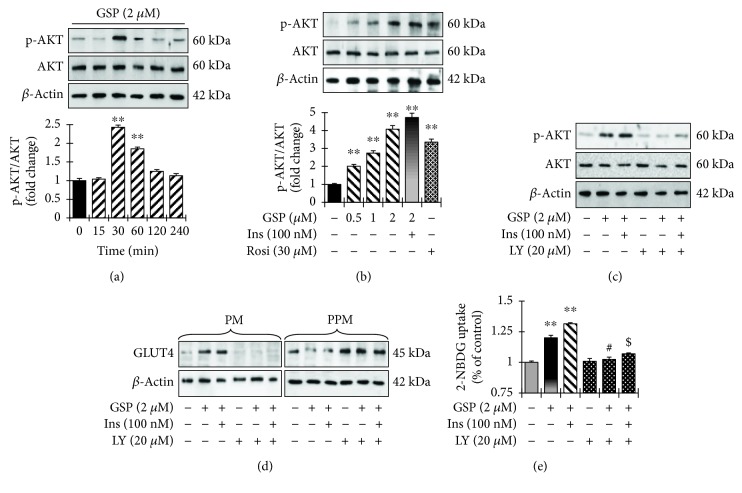
Involvement of the Akt-signaling pathway in gossypol- (GSP-) induced stimulation of glucose uptake. (a) Time course of the effect of GSP on AKT phosphorylation in C2C12 myotubes. Pretreatment of cells with GSP (2 *μ*M) and then incubated with indicated time periods. Cell lysates were prepared, and immunoblotting was performed. Statistical data are represented in the adjacent figure. Values denote means ± SEM of three independent experiments. ^∗∗^ *p* < 0.01 versus no treatment. (b) C2C12 myotubes were incubated with different concentrations of GSP alone for 2 h, or GSP (2 *μ*M) for 2 h followed by insulin (100 nM) for 30 min. Cell lysates were separated by SDS-PAGE, and immunoblotting was carried out. Statistical data are represented in the adjacent figure. Values denote means ± SEM of three independent experiments. ^∗∗^ *p* < 0.01 versus no treatment. (c) Effects of Akt inhibitor LY294002 on Akt-signaling pathway. (d) Effects of LY294002 on the translocations of GLUT4 induced by GSP in C2C12 myotubes. Subcellular membrane fractions were extracted and subjected to immunoblot analysis. (e) Effect of GSP on glucose uptake in LY294002-pretreated C2C12 myotubes. Values denote means ± SEM in independent triplicate. ^∗∗^ *p* < 0.01 versus basal glucose uptake (no stimulation by insulin); ^#^ *p* < 0.05 versus compound-treated basal glucose uptake; ^$^ *p* < 0.01 versus compound-treated insulin-induced glucose uptake.

We used RT-PCR and Western blotting analysis to determine whether GSP increases glucose uptake by activating insulin signaling and the expression of insulin receptor (IR*β*), insulin receptor substrate (IRS-1), and 3-phosphoinositide-dependent protein kinase 1 (PDK1). As expected, insulin stimulation augmented the transcriptional and translational levels of IR*β*, IRS-1, and PDK-1 (Figures 5(a) and 5(b)). GSP treatment also significantly enlarged the transcriptional and translational levels of IRS-1 and PDK1 in a concentration-dependent manner (Figures 5(a) and 5(b)). However, GSP alone did not affect the level of IR*β* phosphorylation (Figure 5(b)). The relative degrees of IR*β*, IRS-1, and PDK1 phosphorylation are presented in Figure 5(c). Moreover, to determine whether GSP treatment also enhances the activation of AKT, a key signaling molecule in insulin-stimulated GLUT4 translocation, we used immunoblotting to measure time-dependent and concentration-dependent AKT phosphorylation levels. As shown in Figure 6(a), GSP treatment augmented AKT phosphorylation at 30 min. Furthermore, GSP significantly enhanced AKT phosphorylation in a concentration-dependent fashion (Figure 6(b)), whereas GSP treatment did not affect on the phosphorylation of AMPK (Supplementary Figure S3). Taken together, the results indicate that GSP significantly activates IRS-1 in the absence of the activation of insulin receptor, resulting in enhanced GLUT4 translocation and glucose uptake.

We used LY294002 (a selective AKT inhibitor) to ascertain whether the phosphorylation of AKT by GSP is participating in the augmentation of GLUT4 translocation to the cell membrane, resulting in activated glucose uptake. LY294002 significantly prevented the phosphorylation of AKT, even subsequent GSP treatment with or without insulin (Figure 6(c)). Furthermore, GLUT4 translocation induced by GSP treatment with or without insulin was significantly attenuated when AKT was blocked in C2C12 myotubes (Figure 6(d)). LY294002 also significantly suppressed the aptitude of GSP to increase glucose uptake. Both basal- and insulin-mediated glucose uptake levels were increased by 2 *μ*M GSP, but were significantly attenuated by LY294002 treatment (by 18.1% and 25.4%, respectively) (Figure 6(e)). The above data advocate that the AKT signal pathway is vital for higher glucose uptake activated by GSP.

### 3.5. *In Vivo* Antidiabetic Activity of GSP in STZ-Stimulated Mice

**Figure 7:**
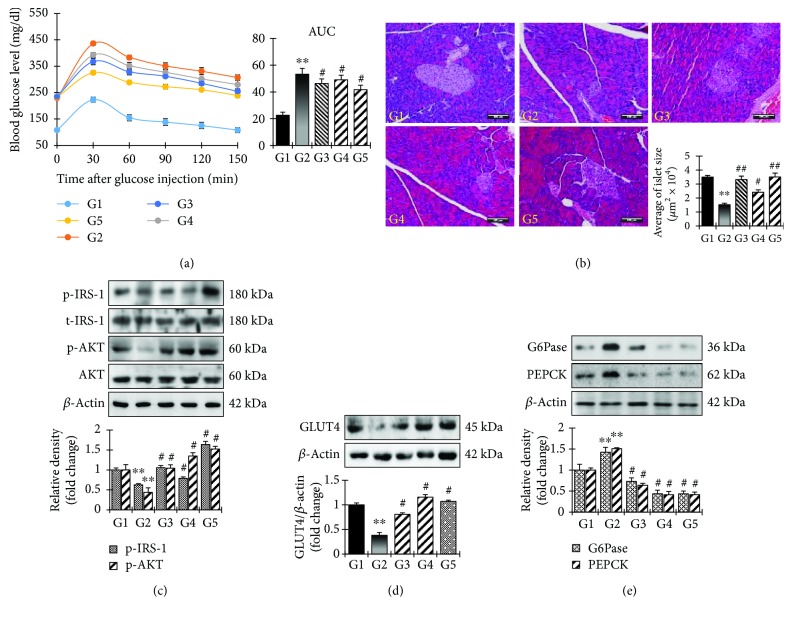
Analysis of glucose homeostasis in STZ-induced diabetic mice by gossypol (GSP). (a) STZ-induced diabetic mice were treated with GSP (1 mg/kg and 2.5 mg/kg) or rosiglitazone at 10 mg/kg, subjected to an oral glucose tolerance test and monitored after an oral load of glucose (1 mg/kg) at different time intervals. (b) Typical photomicrographs of the hematoxylin and eosin-stained pancreases of STZ-induced diabetic mice. (c, d) After the treatment, skeletal muscle tissues were excised and Western blotting analysis was performed. Statistical data are represented in the adjacent figure. Values are shown as means ± SEM in triplicate. ^∗∗^ *p* < 0.01 versus no treatment (G1), ^#^ *p* < 0.05 versus STZ-control (G2). (e) Effect of GSP on gluconeogenesis enzymes. After the treatment, liver tissues were excised and Western blotting analysis for gluconeogenesis enzymes was performed.

Having determined that GSP significantly increases glucose uptake in C2C12 cells, we examined its effects on the control of hyperglycemia in STZ-induced diabetic mice. The GSP treatment groups (G4 and G5) exhibited significantly reduced blood glucose levels post glucose load (1 g/kg) in the STZ-induced diabetic mice (Figure 7(a)). Histopathological examinations revealed normal acini and normal cellularity in the islets of Langerhans of the control pancreases (G1, Figure 7(b)). There was extensive damage to the islets of Langerhans and a reduced number of islet cells in the diabetic control group (G2, Figure 7(b)). Diabetic animals treated with 2.5 mg/kg GSP (G5, Figure 7(b)) had islet cells, normal acini cells, and few pancreatic *β* cell regeneration foci. We used Western blotting to investigate *in vivo* GSP insulin mimicry by examining Irs-1 phosphorylation and Akt expression. As expected, there was significant IRS-1 expression and activation of the downstream Akt signaling pathways in the GSP treatment group (Figure 7(c)). Western blotting analysis also revealed the expected expression of GLUT4 (Figure 7(d)), validating our *in vitro* findings.

GSP can also mitigate oxidative stress in STZ-induced diabetic animal models. Interestingly, antioxidant enzyme expression was completely mitigated in the STZ-induced diabetic control group G2, whereas GSP treatment boosted the expression of both phase I and phase II antioxidant enzymes in the livers of the STZ-induced diabetic animal models (Supplementary Figure S4). These results recommended that the antioxidant capacity of GSP may aid the uptake of glucose by skeletal muscles and lessen blood glucose levels in STZ-induced diabetic models. We also scrutinized the mRNA and protein expression levels of phosphoenolpyruvate carboxykinase (PEPCK) and glucose 6-phosphatase (G6Pase) in the mouse livers to assess the effects of GSP on gluconeogenesis in STZ-induced diabetic mice. PEPCK and G6Pase mRNA and protein expression levels increased in the GSP treatment groups, whereas they were strongly mitigated in the STZ-induced diabetic groups (Figure 7(e); Supplementary Figure S5). This suggests that GSP helped to ameliorate hyperglycemia by abolishing gluconeogenesis in the STZ-induced diabetic mice.

## 4. Discussion

DM is a perplexing metabolic disease that has serious health implications. Insulin, insulin secretagogues, insulin sensitizers, and prandial glucose regulators are used both individually and in combination to attain better glycemic regulation. However, many of these drugs can retain severe adverse effects in patients with indigent tolerance [17]. Therefore, more active hypoglycemic agents with fewer side effects are needed. Mounting evidence suggests that many natural products are potentially useful for treating DM, and they are considered an important resource in the development of DM therapies [18, 22, 23]. In the present study, we discovered that gossypol (GSP) significantly inhibited *α*-glucosidase activity in a reversible, competitive, concentration-dependent manner. Furthermore, GSP treatment enhanced glucose uptake by activating insulin-mediated signaling and augmented GLUT4 translocation, both *in vitro* and *in vivo*. This suggests that GSP has a potential in controlling glucose homeostasis as a novel hypoglycemic agent for the treatment of T2-DM.

*α*-Glucosidase is a strategic enzyme that metabolizes nonabsorbable oligosaccharides into absorbable monosaccharides in the small intestine. Suppression of this enzyme can interrupt the conversion of oligosaccharides and disaccharides into monosaccharides, lessening glucose absorption and subsequently dropping postprandial hyperglycemia. Therefore, *α*-glucosidase inhibition is a useful strategy for investigating the effects of nutraceuticals on T2-DM [24–26]. However, there is very little in-depth information about the mechanisms by which *α*-glucosidase is inhibited. In the present study, Lineweaver–Burk plots revealed that GSP inhibited *α*-glucosidase in a competitive manner; at 4 *μ*M, the Km was 9.28 × 10^−4^ mM and the *V*_max_ was 14.29 × 10^−2^ (Δ*A*_405_ per min).

GLUT4 is one of the 14-member GLUT/SLC2A family of facilitative transmembrane hexose transporters and is widely distributed in skeletal muscle, the myocardium, fatty tissue, the kidney, and the brain. GLUT4, which is transferred from GLUT4 vesicles in the cytoplasm to the cell membrane by the action of insulin or muscle contraction, plays a linchpin role in the transport of glucose into the cells [27, 28]. The reduction of GLUT4 translocation is one of the most substantial causes of insulin resistance in T2-DM [29]. In the present study, we uncovered that GSP drastically amplified glucose uptake through augmentation of GLUT4 expression and translocation in C2C12 cells (Figure 4). GSP may be effective at alleviating insulin resistance in T2-DM.

We sought to ascertain the molecular mechanism underlying the augmentation of GLUT4 expression and translocation. There is accumulating studies suggesting that GLUT4 expression and translocation are induced by insulin or exercise, leading to the promotion of glucose uptake [22]. In the insulin pathway, the activated form of InsR by insulin causes the phosphorylation of IRS tyrosine residues, and PDK1, guiding to the activation of PI3K and its downstream molecules such as AKT, which stimulate GLUT4 translocation [30]. AICAR and metformin (muscle contraction and/or the AMPK activators) activate the AMPK pathway by elevating the AMP to ATP ratio in skeletal muscle [31]. In this study, we discovered that GSP activated the phosphorylation of IRS-1, PDK1, and AKT, but did not affect IR*β* or AMPK. Furthermore, to confirm whether GSP enhances glucose uptake through the activation of AKT signaling, we used LY294002, a specific AKT inhibitor. As shown in Figure 6, LY294002 significantly abolished the translocation of GLUT4 and the phosphorylation of the AKT molecule. LY294002 treatment also strongly attenuated basal- and insulin-stimulated glucose uptake, indicating that AKT and IRS-1 downstream molecules are involved in GSP-induced insulin signaling. However, unlike insulin, GSP stimulated IRS-1 independently of insulin receptor activation. This result suggests that IR inhibition does not significantly alter GSP-induced glucose transport, but further research is needed to reveal how GSP activates the IRS-1/AKT pathway. Although insulin binds with IRs and stimulates the insulin signaling pathway, the action of insulin can be mimicked without the activation of IRs [32]. Dehydroepiandrosterone (DHEA) has been shown to augment glucose transport via stimulation of the translocation of GLUT4 through the activation of the IRS-1/Akt signaling pathway in adipocytes, without activating IRs. The authors speculated that DHEA binds to specific G-protein receptors on the cell surface to activate the IRS-1/PI3K pathway [33, 34]. A racemic mixture of GSP can also exist in various symmetrical or asymmetrical tautomeric forms, resulting in varying lipid solubility. Moreover, GSP can form a Schiff base when its aldehyde groups react with the amino groups of the lysine residues on enzymes, or via H-bond formation with the catechol hydroxyls, which alters enzyme function [2]. Therefore, we hypothesize that GSP may initiate the insulin signaling pathway by activating the IRS-1/Akt pathway, boosting the translocation of GLUT4 by activating other membrane receptors. This requires further investigation.

Diabetes is associated with oxidative stress/damage, which can lead to the glycation of tissue proteins and glucose auto-oxidation. This results in the generation of reactive oxygen species (ROS) and protein-reactive ketoaldehydes, which leads to increased lipid peroxidation and oxidative DNA damage [35]. Moreover, the formation of advanced glycation end products (AGEs) activates transcription factor nuclear factor kappa B (NF-*κ*B) and its various downstream gene targets, leading to the overproduction of nitric oxide (NO), which is believed to be mediator of *β*-cell damage [18]. Thus, antioxidants can provide defense against oxidative stress and have beneficial implications for diabetes management. In the present study, phase I antioxidant enzymes such as CAT, GPx1, and SOD1 and phase II antioxidant enzymes such as HO-1 were mitigated in the G2 group compared to the G1 group, possibly owing to enhanced glucose oxidation, AGE-mediated free radical generation, or the action of STZ as an NO donor [36]. The oral administration of GSP boosted CAT, GPx1, HO-1, and SOD1 protein expressions in diabetic mice livers (Supplementary Figure S4). This suggests that GSP has strong antioxidant activity and can quench free radicals and is therefore able to prevent the complications associated with diabetes.

Hepatic gluconeogenesis is required to maintain the homeostasis of blood glucose during fasting and is the main contributor to postprandial and fasting hyperglycemia in diabetes [37]. It is worth noting that the abandoned expression and activity of gluconeogenic enzymes increases gluconeogenesis in diabetes. Normally, insulin can mitigate gluconeogenesis through multiple mechanisms. One such mechanism is the direct inhibition of the transcription of key gluconeogenic genes, such as PEPCK and G6Pase, by blocking the recruitment of the transcriptional coactivators PGC-1*α* and CREB-binding protein to the promoters of the PEPCK and G6Pase genes [38]. In the current study, GSP reduced the expressions of PEPCK and G6Pase in the liver compared to the STZ-induced diabetic control group (Figure 7(e)). These findings also revealed that GSP has strong insulin mimetic activity and decreases the blood glucose level. It also enhances the translocation of GLUT4 and mitigates gluconeogenesis in the livers of diabetic mice, possibly through the activation of AKT, but further research on the mechanism involved is required.

## 5. Conclusion

**Figure 8:**
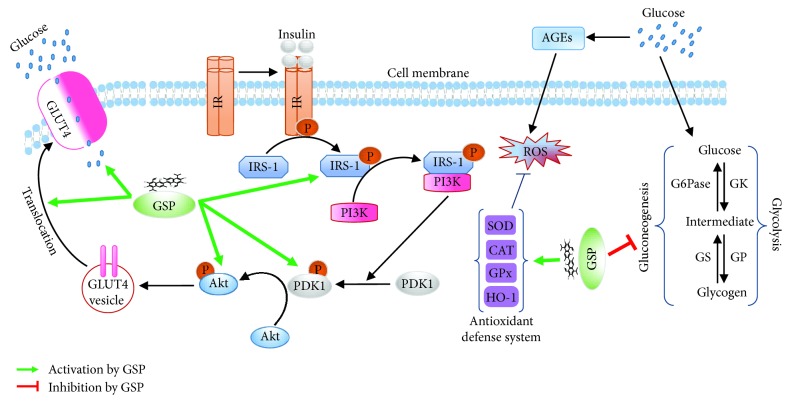
A proposed mechanism of action of gossypol (GSP) in glucose homeostasis through IRS-1/Akt signaling cascade activation and gluconeogenesis repression.

The present study revealed that GSP strongly inhibits *α*-glucosidase activity in a competitive manner. GSP also accelerates glucose transport in C2C12 myotubes by inducing the translocation of GLUT4 via an insulin-mimicking signaling pathway. In an *in vivo* study, GSP significantly amended oral glucose tolerance in an STZ-induced diabetic mouse model. GSP treatment boosted GLUT4 expression through the phosphorylation of AKT in muscle tissue and attenuated the gluconeogenesis pathway by downregulating glucose-6-phosphatase (G6Pase) and phosphoenolpyruvate carboxykinase (PEPCK) in the liver (Figure 8). These findings may improve our understanding of the hypoglycemic and antidiabetic effects of GSP.

## Figures and Tables

**Table 1:** Kinetic parameters of *α*-glucosidase in the presence of gossypol (GSP).

Compound	Concentration (mM)	Km (mM)	*V*_max_ (Δ*A*_405_ per min)	Mode of inhibition
None	—	2.26 × 10^−4^	14.26 × 10^−3^	
GSP	1 × 10^−3^	3.04 × 10^−4^	14.11 × 10^−3^	Competitive
	2 × 10^−3^	4.15 × 10^−4^	14.26 × 10^−3^	
	3 × 10^−3^	6.00 × 10^−4^	14.31 × 10^−3^	
	4 × 10^−3^	9.28 × 10^−4^	14.29 × 10^−3^	

## Data Availability

The data used to encourage the findings of this study are obtainable from the corresponding author upon request.

## Supplementary Materials

Supplementary Materials Table S1: list of the primer sets used in the study. Supplementary Figure S1: HPLC profile of gossypol in cotton seed extracts. Supplementary Figure S2: effect of gossypol (GSP) on the viability of C2C12 myotubes. Supplementary Figure S3: effect of GSP on the phosphorylation of AMPK in C2C12 myotubes. Supplementary Figure S4: effect of GSP on antioxidant enzymes. Supplementary Figure S5: effect of GSP on gluconeogenesis enzymes.
