# Supplementary material for: Gossypol from Cottonseeds Ameliorates Glucose Uptake by Mimicking Insulin Signaling and Improves Glucose Homeostasis in Mice with Streptozotocin-Induced Diabetes
Source: Oxid Med Cell Longev. 2018 Oct 28;2018:5796102. doi: 10.1155/2018/5796102 (PMC6230386; doi:10.1155/2018/5796102)
Supplement: Supplementary Materials — Table S1: list of the primer sets used in the study. Supplementary Figure S1: HPLC profile of gossypol in cotton seed extracts. Supplementary Figure S2: effect of gossypol (GSP) on the viability of C2C12 myotubes. Supplementary Figure S3: effect of GSP on the phosphorylation of AMPK in C2C12 myotubes. Supplementary Figure S4: effect of GSP on antioxidant enzymes. Supplementary Figure S5: effect of GSP on gluconeogenesis enzymes. [file 5796102.f1.docx]

**Gossypol from cottonseeds ameliorates glucose uptake by mimicking insulin signaling and improves glucose homeostasis in mice with streptozotocin-induced diabetes**

Md Badrul Alam^1,$^, Hongyan An^1,$^, Jeong-Sic Ra^1^, Ji-young Lim^1^, Seung-Hyun Lee^1^, Chi-Yeol Yoo^1^, Sang-Han Lee^1,*^

^1^Department of Food Science and Biotechnology, Graduate School, Kyungpook National University, Daegu 41566, Republic of Korea

**^$^**These authors made equal contributions

*Correspondence to:

Dr. Sang-Han Lee; Email: sang@knu.ac.kr; Tel: +82-53-950-7754

**Supporting Information**

**2 Materials and Methods**

**2.1 Drugs and chemicals**

Gossypol from cottonseed, 𝛼-glucosidase, p-nitrophenyl-𝛼-d-glucopyranoside (pNPG), 3-(4,5-dimethylthiazol-2-yl)-2,5-diphenyltetrazolium bromide (MTT), LY294002 (AKT inhibitor), and dimethyl sulfoxide (DMSO) were purchased from Sigma-Aldrich (St. Louis, MO, USA). Dulbecco’s modified Eagle’s medium (DMEM), fetal bovine serum (FBS), a penicillin–streptomycin mixture, and 0.25% trypsin-ethylenediaminetetraacetic acid (EDTA) were purchased from GE Healthcare Life Sciences (Hyclone, Mordialloc, VIC, Australia). 2-NBDG was purchased from Thermo Fisher Scientific Inc. (Carlsbad, CA, USA). Antibodies—including anti-phospho AKT, anti-AKT, anti-phospho IRβ, anti-IRβ, anti-phospho-IRS1, anti-IRS1, anti-phospho PDK1, anti-PDK1, anti-glucose transporter type 4 (GLUT4), and anti-𝛽-actin—were purchased from Bioworld Technology Inc. (St. Louis Park, MN, USA).

**2.2 MTT assay**

The tetrazolium dye colorimetric test (MTT) was used to determine the viability of C2C12 cells^[1]^. C2C12 cells were first cultured in 96-well plates (1 × 10^5^ cells/well) for 24 h. After reaching 90% confluence, the cells were treated with various concentrations of GSP. After 24 h of incubation, the MTT reagent was added to each well, and the plate was incubated at 37°C for 1 h. The media were removed and the wells were washed twice with PBS (pH 7.4). The intracellular insoluble formazan was dissolved in 100% DMSO. The absorbance in each well was measured at 570 nm using a microplate reader (Perkin Elmer, Wallac Victor3, MA, USA), and the viability percentage was calculated.

**2.3 High-performance liquid chromatography (HPLC) analysis**

The presence of gossypol in the cottonseeds was confirmed by high-performance liquid chromatography (HPLC) using gossypol as a standard. HPLC analysis was carried out using an Agilent 1200 chromatographic system (Agilent Technologies, Santa Clara, CA, USA) with a UV-Vis diode-array detector and ChemStation software (version G2170BA, Agilent Technologies). The samples were filtered through a 0.45-μm nylon filter (E0034, Análisis Vínicos, Tomelloso, Spain), and polyphenolic compounds were analyzed according to the method described by Hron et al.^[2]^. A MetaChem Technologies Inc (Torrance, CA) Inertsil (5 μ, ODS-3, 100 x 3.0 mm) reversed-phase column was maintained at 30°C, and elution was performed isocratically with a mobile phase flow rate of 1.0 mL/min. The mobile phase consisted of 80% acetonitrile and 20% 10 mM KH_2_PO_4_ buffer adjusted to pH 3.0 with H_3_PO_4_. The gossypol in the cottonseed was identified by comparison of the retention times with those of the gossypol standard.

**2.4 Blinded experiment**

The *in vitro* experimenters were not blinded to group assignment or outcome assessment. However, the treatment of samples and animals, and the assessment of results were blinded to minimize subjective bias, and no data were excluded from the experimental results.

**Table S1: List of the primer sets used in the study**

| ***Gene name*** |  | ***Sequences*** |
| --- | --- | --- |
| *GLUT4* | *forward* | *GTCCAATGTCCTTGCTCCAG* |
|  | *reverse* | *CGTCGTCCAGCTCGTTCTAC* |
| *Ir* | *forward* | *GAGATGGTCCACCTGAAGGA* |
|  | *reverse* | *ATCAGGTTCCGAACAGTTGC* |
| *IRS-1* | *forward* | *AAGCACCTGGTGGCTCTCTA* |
|  | *reverse* | *TCAGGATAACCTGCCAGACC* |
| *PDK1* | *forward* | *GGGCCAACTCATTTGTAGGA* |
|  | *reverse* | *CCCTTCCATCTCTTCACAGC* |
| *PEPCK* | *forward* | *AACACACCCTCGGTCAACAG* |
|  | *reverse* | *GATATACTCCGGCTGGCACA* |
| *FBP1* | *forward* | *ATGCTGAAGTCGTCCTACGC* |
|  | *reverse* | *ACTGCCATAGAGCGCATACC* |
| *G6PC* | *forward* | *CTGTGCAGCTGAACGTCTGT* |
|  | *reverse* | *GCTGGCAAAGGGTGTAGTGT* |
| *Gapdh* | *forward* | *TTGTGATGGGTGTGAACCAC* |
|  | *reverse* | *ACACATTGGGGGTAGGAACA* |

**Supporting Information Figures**

**
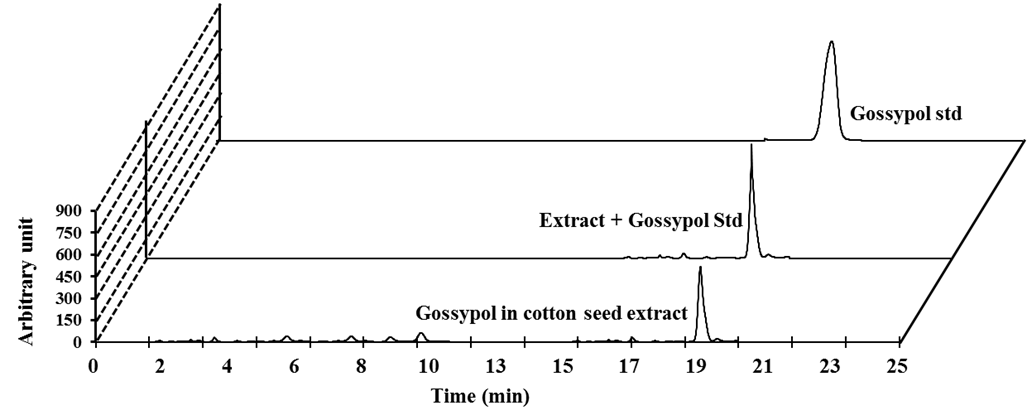
**

**Figure S1. HPLC profile of gossypol in cotton seed extracts.**

**
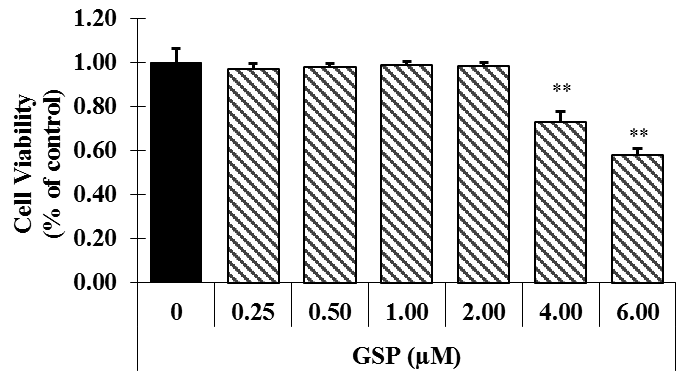
**

**Figure S2. Effect of gossypol (GSP) on the viability of C2C12 myotubes.** Cells were treated with GSP for 24 h at the indicated concentrations, and cell viability was determined by MTT assay. Values are expressed as the mean ± SD (n = 3).

**
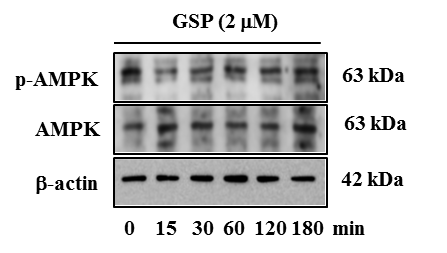
**

**Figure S3. Effect of GSP on the phosphorylation of AMPK in C2C12 myotubes.** C2C12 myotubes were incubated with various concentrations of GSP alone for 2 h, or GSP (2 μM) for 2 h followed by insulin (100 nM) for 30 min. Cell lysates were resolved by SDS-PAGE, and immunoblotting was performed.


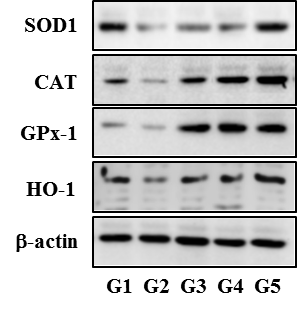


**Figure S4. Effect of GSP on antioxidant enzymes.** STZ-induced diabetic mice were treated with a 10 mg·kg^-1^ dose of GSP or rosiglitazone, and after the treatment liver tissue was excised using RIPA buffer. Western blotting analysis for antioxidant enzymes was performed. Normal control (G1); streptozotocin (STZ)-induced diabetic control (G2); STZ-induced diabetic plus rosiglitazone (10 mg·kg^-1^ b.w.) (G3); STZ-induced diabetic plus low-dose GSP (1 mg.kg^-1^ b.w.) (G4); and STZ-induced diabetic plus high-dose GSP (2.5 mg.kg^-1^ b.w.) (G5).


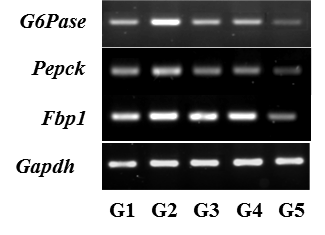


Figure S5. **Effect of GSP on gluconeogenesis enzymes.** STZ-induced diabetic mice were treated with a 10 mg.kg^-1^ dose of GSP or rosiglitazone. After treatment, liver tissue was excised, and RT-PCR analysis of gluconeogenesis enzymes was performed. Normal control (G1); streptozotocin (STZ)-induced diabetic control (G2); STZ-induced diabetic plus rosiglitazone (10 mg·kg^-1^ b.w.) (G3); STZ-induced diabetic plus low-dose GSP (1 mg.kg^-1^ b.w.) (G4); and STZ-induced diabetic plus high-dose GSP (2.5 mg.kg^-1^ b.w.) (G5).

**References**

[1] Zhao, P., Alam, M. B., Lee, S. H., Kim, Y. J., Lee, S., An, H., Choi, H. J., Son, H. U., Park, C. H., Kim, H. H., & Lee, S. H. (2017). Spatholobus suberectus Exhibits Antidiabetic Activity In Vitro and In Vivo through Activation of AKT-AMPK Pathway. Evid Based Complement Alternat Med, 2017, 6091923.

[2] Hron Sr. R. J., Kim H. L., Calhoun M. C., Fishera, G. S. (1999). Determination of (+)-, (−)-, and Total Gossypol in Cottonseed by High-Performance Liquid Chromatography. J. Am. Oil Chem. Soc., 76, 1351–1355.
